# Cerebrospinal Fluid Space Alterations in Melancholic Depression

**DOI:** 10.1371/journal.pone.0038299

**Published:** 2012-06-28

**Authors:** Esther Via, Narcís Cardoner, Jesús Pujol, Ignacio Martínez-Zalacaín, Rosa Hernández-Ribas, Mikel Urretavizacaya, Marina López-Solà, Joan Deus, José Manuel Menchón, Carles Soriano-Mas

**Affiliations:** 1 Department of Psychiatry, Bellvitge University Hospital-IDIBELL, Barcelona, Spain; 2 Carlos III Health Institute, Centro de Investigación Biomédica en Red de Salud Mental (CIBERSAM), Spain; 3 Department of Clinical Sciences, School of Medicine, University of Barcelona, Barcelona, Spain; 4 CRC Hospital del Mar, Barcelona, Spain; 5 Department of Clinical and Health Psychology, Autonomous University of Barcelona, Barcelona, Spain; Chiba University Center for Forensic Mental Health, Japan

## Abstract

Melancholic depression is a biologically homogeneous clinical entity in which structural brain alterations have been described. Interestingly, reports of structural alterations in melancholia include volume increases in Cerebro-Spinal Fluid (CSF) spaces. However, there are no previous reports of CSF volume alterations using automated whole-brain voxel-wise approaches, as tissue classification algorithms have been traditionally regarded as less reliable for CSF segmentation. Here we aimed to assess CSF volumetric alterations in melancholic depression and their clinical correlates by means of a novel segmentation algorithm (‘new segment’, as implemented in the software Statistical Parametric Mapping-SPM8), incorporating specific features that may improve CSF segmentation. A three-dimensional Magnetic Resonance Image (MRI) was obtained from seventy patients with melancholic depression and forty healthy control subjects. Although imaging data were pre-processed with the ‘new segment’ algorithm, in order to obtain a comparison with previous segmentation approaches, tissue segmentation was also performed with the ‘unified segmentation’ approach. Melancholic patients showed a CSF volume increase in the region of the left Sylvian fissure, and a CSF volume decrease in the subarachnoid spaces surrounding medial and lateral parietal cortices. Furthermore, CSF increases in the left Sylvian fissure were negatively correlated with the reduction percentage of depressive symptoms at discharge. None of these results were replicated with the ‘unified segmentation’ approach. By contrast, between-group differences in the left Sylvian fissure were replicated with a non-automated quantification of the CSF content of this region. Left Sylvian fissure alterations reported here are in agreement with previous findings from non-automated CSF assessments, and also with other reports of gray and white matter insular alterations in depressive samples using automated approaches. The reliable characterization of CSF alterations may help in the comprehensive characterization of brain structural abnormalities in psychiatric samples and in the development of etiopathogenic hypotheses relating to the disorders.

## Introduction

Melancholic depression is a subtype of major depressive disorder (MDD) that encompasses a constellation of distinctive clinical features such as anhedonia, distinct quality of mood and mood non-reactivity, psychomotor disturbances, feelings of guilt, early awakening, diurnal variation and anorexia [1]–[4]. Specific neurobiological correlates such as cortisol dysregulation and altered sleep patterns have also been appreciated in melancholia [5]–[7]. Indeed, since all these symptoms are regularly present in most melancholic patients, melancholia may be considered as a biologically homogeneous clinical entity, especially when compared with other depression subtypes [2], [8].

In addition to such features, a number of studies have also identified brain structural alterations in melancholia [9]–[15], such as volume reductions in the hippocampus [9], right anterior supplementary motor area [14] or left insula [15]. Interestingly, in a subset of these studies [10]–[12], alterations in cerebro-spinal fluid (CSF) spaces were also described. Thus, using Region-of-Interest (ROI) approaches, early reports from our group, and other groups, showed volumetric enlargements of the CSF spaces surrounding the upper frontal lobe [12] and the left Sylvian fissure [10], [11]. Furthermore, in one of the studies [10], CSF increases in the left Sylvian fissure were related to the time to remission of the depressive episode, thus giving clinical relevance to the findings.

Voxel-wise methods such as voxel based morphometry (VBM) [16] are particularly appropriate for the study of brain structural alterations as they offer an unbiased estimation of whole-brain abnormalities [17]. However, since VBM strongly depends on accurate brain tissue segmentation [17], CSF alterations have rarely been assessed using such procedures as CSF segmentation has traditionally been regarded as a rather unreliable approach. Indeed, although in most segmentation algorithms CSF is accurately isolated from gray and white matter, it is not uncommon for segmented CSF images to include voxels from non-brain structures such as the dura, the venous sinuses, the scalp or the skull. Consequently, until now we have not attempted to directly replicate with a voxel-wise technique the CSF findings previously described in melancholic samples using ROI approaches.

Nevertheless, the development of new tissue segmentation algorithms, such as the so-called ‘new segment’ algorithm [18] (included in the last version of the Statistical Parametric Mapping software, SPM8 [19]), may help to overcome such limitations, as it provides further information as to the *a priori* distribution of non-brain tissue. The new algorithm should prevent misclassification of non-brain voxels as CSF [18]. The aim of this study was to assess whole-brain voxel-wise alterations in the CSF spaces of a sample of melancholic patients in comparison to a group of control subjects of similar age and gender distribution. To evaluate the benefits of using the ‘new segment’ algorithm, we compared the results obtained using such a method with those obtained by means of the ‘unified segmentation’ approach [20], as implemented in both SPM5 and SPM8. In addition, the most relevant between-group differences observed with the ‘new segment’ algorithm were validated by means of a non-automated ROI analysis. Finally, to evaluate the relevance of CSF alterations, findings were correlated with the clinical data of the study sample.

## Methods

### Ethics Statement

The study protocol was approved by the ethical committee of clinical research (CEIC) of the Bellvitge University Hospital, and was in compliance with the national legislation and the principles expressed in the Declaration of Helsinki. All participants gave written informed consent after detailed description of the study.

### Subjects

A total of 70 inpatients (41 women, mean age ± SD = 61.56±9.68) with melancholic depression were consecutively recruited from the Mood Disorders Unit of the Bellvitge University Hospital, Barcelona. Sample characteristics are shown in Table 1 and have been reported elsewhere [15]. Patients included in the study met criteria for a current major depressive episode with melancholic features according to DSM IV-TR criteria [1]. Diagnosis was independently confirmed by two senior psychiatrists (M.U. and N.C.) using the Structured Clinical Interview for DSM IV-TR Axis I Disorders–Clinician Version [21]. MDD severity was assessed by means of the Hamilton Rating Scale for Depression-17 items [22] (HAM-D), in which all patients scored between moderate and severe depressive episodes (HAM-D range between 18 and 51 points). The control group was made up of 40 subjects (23 women, mean age ± SD = 59.23±7.09) of similar age and gender distribution and from the same socio-demographic environment.

**Table 1:** Clinical characteristics of melancholic patients.

Age at onset of MDD, years; mean ± SD (range)	51.11±12.57 (17–78)
Number of previous depressive episodes; mean ± SD (range)	3.07±4.13 (1–27)
Duration of Illness, years; mean ± SD (range)	10.45±10.08 (0–40)
Time to remission, days; mean ± SD (range)	58.48±46.82 (8–180)
Treatment duration at inclusion, days; mean ± SD (range)	999.39±1355.76 (0–7300)
HAM-D 17 at admission; mean ± SD (range)	28.60±7.63 (18–51)
HAM-D 17 percentage of reduction at discharge; mean ± SD (range)	85.52±13.12 (30–100)
Treatment status (>4weeks) at time of MRI; n (%)	
Medication-Free	20 (28.6)
Imipramine	17 (24.3)
SSRI	10 (14.3)
Venlafaxine	3 (4.3)
Clomipramine	2 (2.9)
Imipramine with Others	7 (10)
SSRI with Others	1 (1.4)
Clomipramine with Others	2 (2.9)
Other antidepressants	8 (11.4)

HAM-D, Hamilton Rating Scale for Depression (17 items); MDD, Major Depressive Disorder; MRI, Magnetic Resonance Imaging; SSRI, Serotonin Selective Reuptake Inhibitors.

A detailed medical history was recorded and a structured interview was administered to detect subjects who fulfilled exclusion criteria. For both groups, exclusion criteria included the presence or past history of any Axis I or Axis II diagnosis (excepting MDD in patients), the presence or past history of neurologic or other serious medical conditions (including dementia), any contraindication to MRI scanning, or abnormal MRI upon visual inspection. Specifically, to ensure there were no cases of cardiovascular etiology, any evidence of ischemic tissue damage in the MRI was regarded as an exclusion criterion. However, subjects with controlled hypertension or diabetes mellitus Type 2 (DM-II) were not excluded (27.59% of the patients and 32.43% of the healthy controls did have either one or both conditions). All patients were clinically followed up for at least two years through contact with the Mood Disorders Unit. During this period, the presence of emerging signs of dementia was ruled out by the routine clinical interview, which included the administration of the Mini-Mental State Examination (MMSE, [23]).

### MRI Acquisition

Subjects were scanned in a 1.5-T scanner (Signa, GE Medical Systems, Milwaukee, WI) to obtain a sixty-slice 3-D SPGR sequence in the axial plane (TR 40 ms, TE 4 ms, pulse angle 30°, field of view 26 cm, matrix size 256×192 pixels, in-plane resolution 1.02 mm^2^, and section thickness 2.5 mm). Imaging data were processed on a Microsoft Windows platform using technical computing software (MATLAB 7.8; The MathWorks Inc, Natick, Mass) and Statistical Parametric Mapping (SPM8; The Wellcome Department of Imaging Neuroscience, London, UK) [19].

### Data Preprocessing

Firstly, images were inspected for the presence of any artifacts preventing further analyses. Preprocessing involved three main steps; tissue segmentation, normalization, and smoothing, which are described below. We used two different segmentation algorithms, but in all cases images were normalized using DARTEL tools [24] and smoothed with an 8 mm full-width at half maximum (FWHM) isotropic Gaussian kernel.

#### Segmentation

Images were segmented in parallel using two different algorithms: the ‘unified segmentation’, as implemented in SPM5 and SPM8 [20], and the ‘new segment’, as implemented in SPM8 [18].

On the one hand, the ‘unified segmentation’ algorithm extracts three tissue classes (gray matter, white matter and CSF) from raw T1 images using a probabilistic framework combining tissue classification, image registration and bias correction. For the purpose of this study, from this step we discarded final output images in normalized space, exclusively keeping files that encoded the segmentation and normalization transformations applied to raw data (the *_seg_sn.mat files). These files were used as an ‘initial import’ in DARTEL tools in order to write out rigidly transformed versions of CSF tissue (rc3*.nii files), which were saved for the subsequent normalization process.

On the other hand, the ‘new segment’ algorithm is an extension of the ‘unified segmentation’ algorithm that uses an extended set of tissue probability maps allowing for a different treatment of voxels outside the brain (i.e., the ones most likely to be classified as CSF). In addition to gray matter, white matter and CSF tissue probability maps, this algorithm incorporates tissue probability maps of bone, soft tissue and air/background distribution. The ‘new segment’ algorithm also segments, normalizes and corrects bias within the same model, and, also as above, we discarded final output images from this preprocessing and kept the rigidly transformed versions of CSF images that were used for DARTEL normalization (rc3*.nii files).

#### Normalization and smoothing

The rigidly transformed images of CSF derived from both the ‘unified segmentation’ and the ‘new segment’ algorithms were normalized using DARTEL [24]. Firstly, with the function ‘create templates’, images were iteratively matched to a template generated from their own mean, so as to generate a series of templates with increasing resolution. Secondly, native space images from CSF were registered to the highest resolution CSF template within a high-dimensional diffeomorphic framework. Spatially normalized tissue maps were then modulated by the Jacobian determinants derived from the corresponding flow-fields to restore the volumetric information lost during the high-dimensional spatial registration.

Finally, bias corrected, tissue segmented, and DARTEL normalized and modulated CSF images were smoothed with an 8 mm FWHM isotropic Gaussian kernel.

### Data Analysis

Global CSF and total intracranial volume (TIV), obtained from segmented images in native space, were compared between groups by means of independent samples t-tests using SPSS (v. 15).

Between-group comparisons of voxel-wise regional volume differences were studied with SPM8 tools. Specifically, two independent sample t-test models (i.e. one for images derived from the ‘unified segmentation’ and one for the images derived from the ‘new segment’ algorithm) were built, with TIV as a nuisance covariate.

Subsequently, in a post-hoc analysis, between-group differences in CSF volume observed in the region of the left Sylvian fissure (see Results section, below) were validated with a non-automated ROI analysis. Specifically, the region was bilaterally traced by one researcher (I.M.Z.), who was blind to subjects’ diagnosis and hemisphere, on 25 consecutive axial slices of the raw T1 image from each participant using MRIcro software [25]. For each hemisphere, we extracted the volume of the ROI (in milliliters) and the mean signal value across the ROI (normalized to global signal intensity and ROI volume). These two measurements contribute to the final voxel values in images used for VBM analyses, since image segmentation relies on raw signal intensity and modulation restores the volumetric information lost after spatial normalization to a template. Data from each hemisphere were entered into SPSS and analyzed by means of independent samples t-tests.

Finally, to study the correlation between CSF alterations and clinical data, we firstly extracted the first eigenvariate from clusters with significant between-group differences. The values were entered into SPSS and correlated (controlling for total intracranial volume) with the number of previous depressive episodes, time to remission of the current episode, HAM-D score at admission and percentage of HAM-D reduction between admission and discharge.

In all the SPM analyses, the significance threshold was set at p<0.05 (family-wise error [FWE] corrected for multiple comparisons), although, for displaying purposes (i.e., figures), we used a threshold of p<0.001 (uncorrected). In SPSS analyses, the significance threshold was set at p<0.05 with the applicable Bonferroni correction when multiple independent analyses were performed (i.e., in correlations between imaging findings and clinical data).

## Results

### Global CSF and Total Intracranial Volumes

Regarding global CSF volume, no significant between-group differences were observed when images were segmented using the ‘new segment’ approach. Nevertheless, a significant global CSF volume increase in melancholic patients was observed when images were segmented using the ‘unified segmentation’ algorithm. By contrast, significant between-group differences in TIV were not observed with either segmentation approach. These results are presented in Table 2.

**Table 2:** Global CSF and Total Intracranial volume in melancholic patients and healthy controls assessed with the different segmentation algorithms.

		Melancholic patients	Healthy controls	Between-groupDifferences	
		Mean ± SD (ml)	Mean ± SD (ml)	t	p
**Global CSF volume**	**New segment**	291.50±36.50	279.60±36.94	1.638	0.104
	**Unified segmentation**	477.26±144.65	406.20±137.76	2.555	**0.012**
**Total Intracranial volume**	**New segment**	1396.07±141.55	1370.74±135.01	0.930	0.355
	**Unified segmentation**	1530.37±213.48	1454.23±176.42	1.912	0.058

ml: Milliliters; SD: Standard Deviation.

### Voxel-wise CSF Assessment

**Figure 1:**
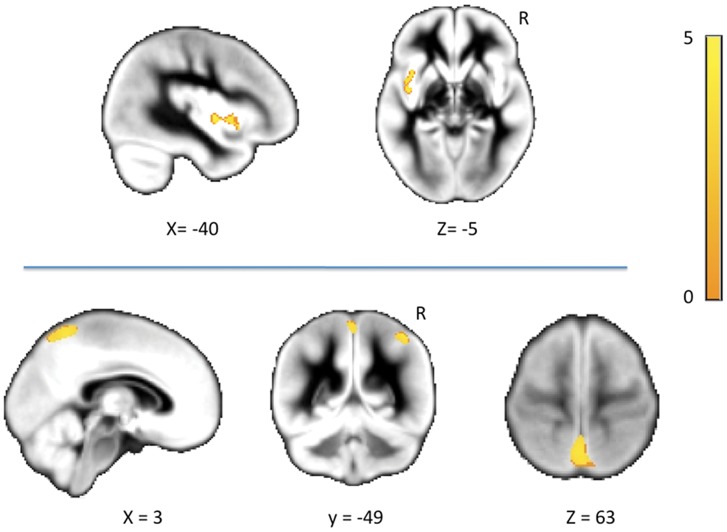
CSF volume alterations in melancholic patients observed using the ‘New Segment’ algorithm. **Upper row.** Left Sylvian fissure CSF increases in melancholic patients. **Lower row.** CSF volume decreases in spaces surrounding the medial and lateral parietal cortices. Voxels with p<0.001 (uncorrected) are overlaid on the study specific DARTEL template. *x* and *z* denote coordinates in DARTEL-template space. The color bar represents t value. R indicates right hemisphere.

Using the ‘new segment’ algorithm, melancholic patients showed a significant CSF volume increase in the region of the left Sylvian fissure surrounding the insular cortex. In addition, we observed a significant volume decrease in the subarachnoid spaces surrounding the medial and the lateral parietal lobe (see Figure 1 and Table 3). Conversely, we found no significant volume changes in regional CSF volumes of melancholic patients when images were segmented using the ‘unified segmentation’ algorithm (between-group differences at the level of the left Sylvian fissure were also detected in terms of a volume increase in melancholic patients, although results were non-significant at the corrected significance threshold: t = 3.51; p_FWE_ = 0.134).

**Table 3:** CSF coordinates of between-group differences using the ‘New Segment’ approach.

	Peak coordinate(x,y,z)*	t value	p value**	Number ofvoxels	Anatomical location
Patients>Controls	**−42 −6 1**	4.46	0.008	18	Left Sylvian fissure
Controls>Patients	**8 −66 57**	4.87	0.002	63	Interhemispheric (medial parietal lobe)
	**39 −54 56**	4.36	0.012	45	Right lateral parietal lobe

* x, y, z denote coordinates in DARTEL-template space.

** FWE corrected for multiple comparisons.

### Validation of Voxel-wise Findings with a ROI Approach

**Figure 2:**
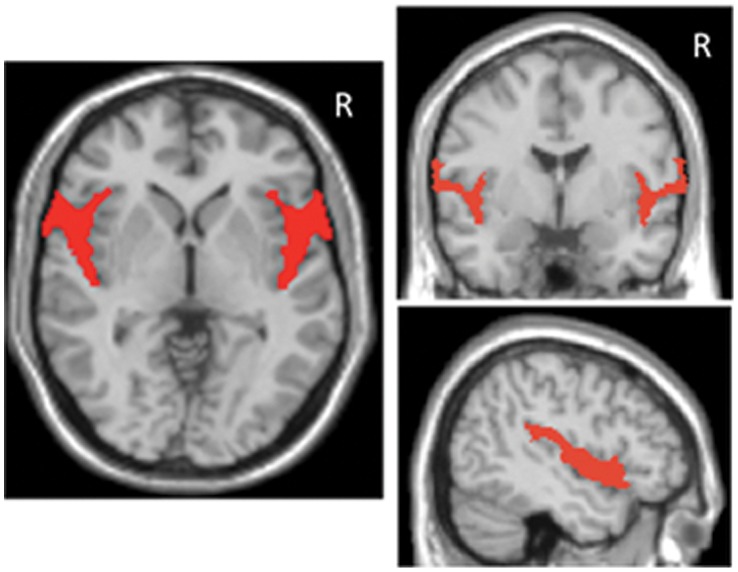
Tracing of the region of the Sylvian fissure overlaid on three orthogonal projections of a normalized brain. R indicates right hemisphere.

To validate our voxel-wise findings, between-group differences in the region of the Sylvian fissure were studied with a ROI approach. This region constitutes an anatomically defined area that may be reliably traced in non-segmented T1 images in native space. The area selected for this analysis is presented in Figure 2 overlaid onto a normalized brain, although for our purposes tracing was performed on individual non-normalized images. Melancholic patients showed lower mean signal intensity across both left and right hemisphere ROIs, which may be interpreted as patients having wider subarachnoidal spaces, resulting in T1 signal intensity from (hypointense) CSF being less affected by partial volume effects from (hyperintense) surrounding gray matter tissue. Likewise, the left hemisphere ROI was larger in melancholic patients, although this was not observed for the right hemisphere region. These results are presented in Table 4.

**Table 4:** Between-group comparison of mean signal intensity and total volume for left and right Sylvian fissure (ROI analysis).

		Melancholic patients	Healthy controls	Between-groupDifferences	
		Mean ± SD	Mean ± SD	t	p
**Mean signal intensity** **(arbitrary units)**	Left	309.90±28.52	335.84±27.97	4.621	0.000
	Right	307.53±27.02	328.29±29.62	3.743	0.000
**ROI volume (ml)**	Left	22.34±3.32	21.10±2.36	2.080	0.040
	Right	20.54±2.75	19.76±2.29	1.531	0.129

ml: Milliliters; SD: Standard Deviation.

### Clinical Correlations

Within the patient group, we correlated CSF volumes from the regions with significant between-group differences in the above analyses with the clinical data. In these analyses a Bonferroni corrected significance threshold was used. Specifically, the threshold was set at p<0.0042 (0.05/12), since we correlated the volume of three different clusters with four clinical variables. CSF volume at the left Sylvian fissure showed a significant negative correlation with the percentage of HAM-D reduction between admission and discharge (r = −0.36; n = 70; p = 0.0040). The same region was also correlated with time to symptom remission after treatment initiation, although this finding was not statistically significant at the Bonferroni corrected significance threshold (r = 0.31; n = 70; p = 0.010). The number of previous depressive episodes and the HAM-D score at admission were not correlated with CSF volume at the left Sylvian fissure. Likewise, CSF volumes surrounding the medial and lateral parietal regions were not correlated with any of the clinical variables assessed.

Finally, in order to assess any medication effects on our results, the number of days of antidepressive treatment at inclusion was correlated against CSF volumes at the regions where between-group differences had been detected in the above analyses, but no significant correlations were found.

## Discussion

To our knowledge, this is the first whole-brain voxel-wise study comparing CSF volumes of melancholic patients with healthy controls. Specifically, we observed a significant CSF increase in the region of the left Sylvian fissure and a CSF volume decrease at the level of the medial and lateral parietal cortex. We also observed that such findings were not detected using earlier segmentation algorithms. By contrast, we validated the left Sylvian fissure alterations by means of a non-automated ROI analysis. Finally, we detected that CSF content in the region of the left Sylvian fissure was inversely related to clinical improvement (i.e., percentage of HAM-D reduction between admission and discharge), which provided our findings with clinical significance.

CSF volume increases in the left Sylvian fissure have been previously reported in other studies with samples of depressive patients [10], [11], [26], [27]. Furthermore, such a finding complements other reports of gray [28]–[30] and white [31] matter volume decreases in the insular cortex of depressive samples. More specifically, findings reported here are in agreement with the reduced gray matter volume in the left insular region reported for the same sample of patients in a previous study by our group [15]. In this sense, results from previous anatomical and functional neuroimaging studies support the idea that the insular cortex is relevantly involved in the pathogenesis of depressive illness. Thus, the insular cortex is closely connected to other limbic components such as the amygdala, ventral striatum and orbitofrontal cortex [32] and it is particularly implicated in the awareness and representation of emotions [32]–[35]. Likewise, data from functional studies in MDD patients have also shown the implication of this area in the abnormal processing of emotions in depression [36]–[39]. Interestingly, lateralization of structural abnormalities to the left insular region has been also reported in previous studies with melancholic samples [11], [29], although the significance of such a finding is not fully clear.

By contrast, significant volume decreases in the CSF spaces surrounding medial and lateral parietal cortices have not been observed in previous studies with melancholic or general depressive samples, in part because this region has not been typically evaluated in region of interest studies. There are, however, some reports of gray matter alterations in medial and lateral parietal areas in depressive patients, both in terms of volume reductions [40]–[42] and increases [43]. Interestingly, Shah et al. [43] reported that gray matter volume increases in the precuneus differentiated between chronic and recovered depressive patients, suggesting that inconsistencies between studies may be partially accounted for by clinical differences between the assessed samples.

In addition to complementing the description of putative structural alterations in gray and white matter, and thus providing a more comprehensive characterization of the structural abnormalities associated to psychiatric conditions, the identification of CSF abnormalities with reliable whole-brain automated approaches may assist the development of etiopathogenic hypotheses relating to the disorders. For example, CSF alterations located in regions with concomitant gray and/or white matter abnormalities may fit within neurodegenerative disease models [44], [45], while larger CSF volumes without gray and/or white matter alterations may relate to neurodevelopmental disturbances, in terms of an abnormal gray/white matter volume increase during early development that is normalized with aging, resulting in larger CSF volumes in adult life, as has been proposed for schizophrenia [46].

According to the above notions, our findings relating to an increased CSF content in the left insular region of melancholic patients, in combination with earlier reports of brain parenchyma reductions in the same region [15], [28]–[31], may be interpreted as neurodegenerative in nature [45]. Conversely, however, the parietal CSF volume reductions reported here are unlikely to be of neurodegenerative origin. Nevertheless, if we consider depression as a chronic and recurrent disease with multiple genetic and environmental determinants [47]–[49], complex models are plausible, and thus structural abnormalities of neurodegenerative origin may well coexist with alterations of another etiology across different brain areas over the course of the illness [15], [44], [45].

In addition to the above ideas, the CSF alterations reported here seem to be of clinical relevance as, in our sample of melancholic patients, larger CSF volumes in the left Sylvian fissure were related to a smaller improvement of depressive symptomatology at discharge. This observation is in accordance with previous correlations obtained with closely related measures, such as regional CSF insular volume [10] or gray matter content of the insular cortex [15], which were related to the time to remission of the depressive episode after treatment initiation. Indeed, we also observed a correlation between CSF volume increase and time to remission, although the result was no longer significant after Bonferroni correction for multiple comparisons. Furthermore, there is also functional evidence supporting the relationship between insular cortex and clinical outcome, such as the observations of a normalization of insular hyperactivity after clinical recovery [39] or successful antidepressant treatment [50], . All in all, such results seem to indicate that the volume of the left insular region may be a relevant predictor of symptom persistence and response to treatment in melancholic depression.

Contrasting the results obtained with the two different image segmentation strategies used here, we observed that the CSF content of the left Sylvian fissure was only significantly increased when data were pre-processed using the ‘new segment’ algorithm. Although there is no ‘gold-standard’ for comparing the results obtained with the two pre-processing approaches, we validated the left Sylvian fissure CSF volume increase detected here under the ‘new segment’ approach with a non-automated ROI analysis. Left, but not right, Sylvian fissure showed a lower mean signal intensity in combination with a larger volume. Also, such a finding is in accordance with earlier studies reporting brain structural alterations in melancholia also using non-automated image analysis techniques [10], [11]. Furthermore, the same CSF increase was also detected using the ‘unified segmentation’ algorithm, albeit at a non-significant level, suggesting a reduced sensitivity of the latter approach to detect regional changes in CSF volume. Parietal CSF volume decreases were also uniquely detected using the ‘new segment’ approach, although, in this case, we did not attempt to replicate the findings with a non-automated ROI approach since findings were located in less definite regions in anatomical terms.

Global CSF measurements also differed between the two segmentation methods studied. While the ‘new segment’ algorithm detected no significant between-group differences, the ‘unified segmentation’ approach showed an increased global CSF volume in melancholic patients. Previous reports showed inconsistent results regarding this measurement, with some studies reporting no changes [52]–[54] and others reporting significant global increases in depressive samples [11], [55]–[58]. Although the differences between studies may partially rely on clinical dissimilarities between samples (i.e., inclusion of patients with late-onset depression [58], or with psychotic symptoms [56]), in the light of our findings, it would seem that global CSF assessments may have been confounded by the inclusion of non-brain tissue. Indeed, in a recent meta-analysis, global CSF increases were not significantly detected in depressive samples and, furthermore, such a measurement was found to depend on methodological factors such as image acquisition parameters [59].

The current study does, however, have certain limitations. Firstly, it is possible that antidepressant treatment may have influenced the reported volumetric measurements, although we did not detect any significant association between our structural measurements and antidepressive treatment time. Secondly, as we did not recruit a sample of non-melancholic MDD patients, our results cannot be considered exclusive to melancholic depression. Such a comparison would be an informative addition to the current findings. Finally, although the ‘new segment’ approach is regarded here as the most valid algorithm to automatically segment CSF content from brain MRIs, further comparisons with other approaches will be required to confirm such an assumption. Nevertheless, the lack of a ‘gold-standard’ protocol for CSF content assessment may perhaps prevent us from obtaining a definite conclusion as to the validity of the different CSF segmentation methods. Be that as it may, voxel-wise alterations located at left Sylvian fissure have been validated with a non-automated ROI analysis, and, moreover, the location of such CSF alterations is not only concordant with earlier reports of regional alterations in the CSF spaces of melancholic samples, but also with previous reports of gray matter alterations in general depressive samples.

In conclusion, this is the first study to show regional (i.e., voxel-wise) CSF volume alterations in melancholic depression using an automated whole-brain segmentation algorithm. Such alterations would seem to have clinical relevance, and may indeed constitute a relevant measurement to be considered in studies seeking imaging predictors of remission or treatment response. Moreover, the description of CSF alterations is necessary for a complete characterization of the neuroanatomical abnormalities of psychiatric conditions, which may help in the development of etiopathogenic hypotheses relating to the disorders. Finally, we have also shown how the characterization of such alterations may depend on the selection of the algorithm used for the automated segmentation of the CSF content from brain images. The results obtained with the ‘new segment’ algorithm, validated here by means of a non-automated ROI assessment, are in agreement with earlier reports also using non-automated methods of CSF segmentation and with findings from similar whole-brain automated methods used for regional gray and white matter volume assessment.

## References

[1] American Psychiatric Association (2000). Diagnostic and Statistical Manual of Mental Disorders.. 4th text revision.Washington, DC.American Psychiatric Association.

[2] Parker G, Fink M, Shorter E, Taylor M, Akiskal H (2010). Issues for DSM-5: Whither melancholia? the case for its classification as a distinct mood disorder.. Am J Psychiatry.

[3] Leventhal AM, Rehm LP (2005). The empirical status of melancholia: Implications for psychology.. Clin Psychol Rev.

[4] Zimmerman M, Coryell W, Pfohl B (1986). Validity of familial subtypes of primary unipolar depression. clinical, demographic, and psychosocial correlates.. Arch Gen Psychiatry.

[5] Taylor MA, Fink M (2006). Melancholia: The Diagnosis, Pathophysiology and Treatment of Depressive Illness.. Cambridge, UK.Cambridge University Press.

[6] Armitage R (2007). Sleep and circadian rhythms in mood disorders.. Acta Psychiatr Scand.

[7] Antonijevic IA (2006). Depressive disorders – is it time to endorse different pathophysiologies?. Psychoneuroendocrinology.

[8] Gold PW, Chrousos GP (2002). Organization of the stress system and its dysregulation in melancholic and atypical depression: High vs low CRH/NE states.. Mol Psychiatry.

[9] Hickie I, Naismith S, Ward PB, Turner K, Scott E (2005). Reduced hippocampal volumes and memory loss in patients with early- and late-onset depression.. Br J Psychiatry.

[10] Cardoner N, Pujol J, Vallejo J, Urretavizcaya M, Deus J (2003). Enlargement of brain cerebrospinal fluid spaces as a predictor of poor clinical outcome in melancholia.. J Clin Psychiatry.

[11] Pujol J, Cardoner N, Benlloch L, Urretavizcaya M, Deus J (2002). CSF spaces of the sylvian fissure region in severe melancholic depression.. Neuroimage.

[12] Baumann B, Bornschlegl C, Krell D, Bogerts B (1997). Changes in CSF spaces differ in endogenous and neurotic depression. A planimetric CT scan study.. J Affect Disord.

[13] Pizzagalli DA, Oakes TR, Fox AS, Chung MK, Larson CL (2004). Functional but not structural subgenual prefrontal cortex abnormalities in melancholia.. Mol Psychiatry 9: 325, 393–405.

[14] Exner C, Lange C, Irle E (2009). Impaired implicit learning and reduced pre-supplementary motor cortex size in early-onset major depression with melancholic features.. J Affect Disord.

[15] Soriano-Mas C, Hernandez-Ribas R, Pujol J, Urretavizcaya M, Deus J (2011). Cross-sectional and longitudinal assessment of structural brain alterations in melancholic depression.. Biol Psychiatry.

[16] Ashburner J, Friston KJ (2000). Voxel-based morphometry–the methods.. Neuroimage.

[17] Ashburner J, Friston KJ (2001). Why voxel-based morphometry should be used.. Neuroimage.

[18] Ashburner J, Barnes G, Chen C, Daunizeau J, Flandin G (2011). New segment. Edited by Functional Imaging Laboratory Wellcome Trust Centre for Neuroimaging Institute of Neurology, UCL, London, UK. In SPM Manual.. http://www.fil.ion.ucl.ac.uk/spm/.

[19] Members & collaborators of the Wellcome Trust Centre for Neuroimaging. SPM software- Statistical Parametric Mapping.. http://www.fil.ion.ucl.ac.uk/spm/software/.

[20] Ashburner J, Friston KJ (2005). Unified segmentation.. Neuroimage.

[21] First MB, Spitzer RL, Gibbon M, Williams JBW (2002). Structured Clinical Interview for DSM-IV-TR Axis I Disorders – Clinician Version (SCID-CV).. Washington, DC: American Psychiatric Publishing.

[22] Hamilton M (1960). A rating scale for depression.. J Neurol Neurosurg Psychiatry.

[23] Folstein MF, Robins LN, Helzer JE (1983). The mini-mental state examination.. Arch Gen Psychiatry.

[24] Ashburner J (2007). A fast diffeomorphic image registration algorithm.. Neuroimage.

[25] Rorden C, Brett M (2000). Stereotaxic display of brain lesions.. Behav Neurol.

[26] Rabins PV, Pearlson GD, Aylward E, Kumar AJ, Dowell K (1991). Cortical magnetic resonance imaging changes in elderly inpatients with major depression.. Am J Psychiatry.

[27] Wurthmann C, Bogerts B, Falkai P (1995). Brain morphology assessed by computed tomography in patients with geriatric depression, patients with degenerative dementia, and normal control subjects.. Psychiatry Res.

[28] Kaufmann C, Kupka E, Nickel T, Zobel A, Puetz B (2001). Grey matter deficits in major depressive episode are unrelated to neuroendocrinologic changes: A voxel-based morphometric analysis of 114 subjects.. NeuroImage.

[29] Takahashi T, Yucel M, Lorenzetti V, Tanino R, Whittle S (2010). Volumetric MRI study of the insular cortex in individuals with current and past major depression.. J Affect Disord.

[30] Peng J, Liu J, Nie B, Li Y, Shan B (2011). Cerebral and cerebellar gray matter reduction in first-episode patients with major depressive disorder: A voxel-based morphometry study.. Eur J Radiol.

[31] Hwang JP, Lee TW, Tsai SJ, Chen TJ, Yang CH (2010). Cortical and subcortical abnormalities in late-onset depression with history of suicide attempts investigated with MRI and voxel-based morphometry.. J Geriatr Psychiatry Neurol.

[32] Craig AD (2011). Significance of the insula for the evolution of human awareness of feelings from the body.. Ann N Y Acad Sci.

[33] Adolphs R, Damasio H, Tranel D, Cooper G, Damasio AR (2000). A role for somatosensory cortices in the visual recognition of emotion as revealed by three-dimensional lesion mapping.. J Neurosci.

[34] Craig AD (2004). Human feelings: Why are some more aware than others?. Trends Cogn Sci.

[35] Critchley HD, Wiens S, Rotshtein P, Ohman A, Dolan RJ (2004). Neural systems supporting interoceptive awareness.. Nat Neurosci.

[36] Davey CG, Allen NB, Harrison BJ, Yucel M (2011). Increased amygdala response to positive social feedback in young people with major depressive disorder.. Biol Psychiatry.

[37] Surguladze SA, El-Hage W, Dalgleish T, Radua J, Gohier B (2010). Depression is associated with increased sensitivity to signals of disgust: A functional magnetic resonance imaging study.. J Psychiatr Res.

[38] Davidson RJ, Irwin W, Anderle MJ, Kalin NH (2003). The neural substrates of affective processing in depressed patients treated with venlafaxine.. Am J Psychiatry.

[39] Mayberg HS, Liotti M, Brannan SK, McGinnis S, Mahurin RK (1999). Reciprocal limbic-cortical function and negative mood: Converging PET findings in depression and normal sadness.. Am J Psychiatry.

[40] Inkster B, Rao AW, Ridler K, Nichols TE, Saemann PG (2011). Structural brain changes in patients with recurrent major depressive disorder presenting with anxiety symptoms.. J Neuroimaging.

[41] Mak AK, Wong MM, Han SH, Lee TM (2009). Gray matter reduction associated with emotion regulation in female outpatients with major depressive disorder: A voxel-based morphometry study.. Prog Neuropsychopharmacol Biol Psychiatry.

[42] Abe O, Yamasue H, Kasai K, Yamada H, Aoki S (2010). Voxel-based analyses of gray/white matter volume and diffusion tensor data in major depression.. Psychiatry Res.

[43] Shah PJ, Ebmeier KP, Glabus MF, Goodwin GM (1998). Cortical grey matter reductions associated with treatment-resistant chronic unipolar depression. Controlled magnetic resonance imaging study.. Br J Psychiatry.

[44] Ballmaier M, Toga AW, Blanton RE, Sowell ER, Lavretsky H (2004). Anterior cingulate, gyrus rectus, and orbitofrontal abnormalities in elderly depressed patients: An MRI-based parcellation of the prefrontal cortex.. Am J Psychiatry.

[45] Savitz J, Drevets WC (2009). Bipolar and major depressive disorder: Neuroimaging the developmental-degenerative divide.. Neurosci Biobehav Rev.

[46] Narr KL, Bilder RM, Woods RP, Thompson PM, Szeszko P (2006). Regional specificity of cerebrospinal fluid abnormalities in first episode schizophrenia.. Psychiatry Res.

[47] Nugent NR, Tyrka AR, Carpenter LL, Price LH (2011). Gene-environment interactions: Early life stress and risk for depressive and anxiety disorders.. Psychopharmacology (Berl).

[48] Kendler KS, Kessler RC, Walters EE, MacLean C, Neale MC (1995). Stressful life events, genetic liability, and onset of an episode of major depression in women.. Am J Psychiatry.

[49] Silberg J, Rutter M, Neale M, Eaves L (2001). Genetic moderation of environmental risk for depression and anxiety in adolescent girls.. Br J Psychiatry.

[50] Kennedy SH, Evans KR, Kruger S, Mayberg HS, Meyer JH (2001). Changes in regional brain glucose metabolism measured with positron emission tomography after paroxetine treatment of major depression.. Am J Psychiatry.

[51] Delaveau P, Jabourian M, Lemogne C, Guionnet S, Bergouignan L (2011). Brain effects of antidepressants in major depression: A meta-analysis of emotional processing studies.. J Affect Disord.

[52] Kumar A, Schweizer E, Jin Z, Miller D, Bilker W (1997). Neuroanatomical substrates of late-life minor depression. A quantitative magnetic resonance imaging study.. Arch Neurol.

[53] Steingard RJ, Renshaw PF, Hennen J, Lenox M, Cintron CB (2002). Smaller frontal lobe white matter volumes in depressed adolescents.. Biol Psychiatry.

[54] Ballmaier M, Sowell ER, Thompson PM, Kumar A, Narr KL (2004). Mapping brain size and cortical gray matter changes in elderly depression.. Biol Psychiatry.

[55] Maller JJ, Daskalakis ZJ, Fitzgerald PB (2007). Hippocampal volumetrics in depression: The importance of the posterior tail.. Hippocampus.

[56] Salokangas RK, Cannon T, Van Erp T, Ilonen T, Taiminen T (2002). Structural magnetic resonance imaging in patients with first-episode schizophrenia, psychotic and severe non-psychotic depression and healthy controls. Results of the schizophrenia and affective psychoses (SAP) project.. Br J Psychiatry.

[57] Pantel J, Schroder J, Essig M, Schad LR, Popp D (1998). Volumetric brain findings in late depression. A study with quantified magnetic resonance tomography.. Nervenarzt.

[58] Pantel J, Schroder J, Essig M, Popp D, Dech H (1997). Quantitative magnetic resonance imaging in geriatric depression and primary degenerative dementia.. J Affect Disord.

[59] Arnone D, McIntosh AM, Ebmeier KP, Munafo MR, Anderson IM (2011). Magnetic resonance imaging studies in unipolar depression: Systematic review and meta-regression analyses. Eur Neuropsychopharmacol.. In Press.

